# From Fear to Hope: Understanding Preparatory and Anticipatory Grief in Women with Cancer—A Public Health Approach to Integrating Screening, Compassionate Communication, and Psychological Support Strategies

**DOI:** 10.3390/jcm14113621

**Published:** 2025-05-22

**Authors:** Jelena Milic, Milica Vucurovic, Edita Grego, Dragana Jovic, Rosa Sapic, Sladjana Jovic, Verica Jovanovic

**Affiliations:** 1 Institute of Public Health of Serbia “Dr Milan Jovanović Batut”, 112113 Belgrade, Serbiavericajovanovic@yahoo.com (V.J.); 2 European Faculty “Kallos”, 11000 Belgrade, Serbia; 3 Faculty of Health Studies, University of Bjeljina, 76300 Bjeljina, Bosnia and Herzegovina; 4 Faculty of Security Studies, University of Belgrade, Gospodara Vučića 50, 11118 Belgrade, Serbia

**Keywords:** preparatory grief, anticipatory grief, psycho-oncology, family systemic psychotherapy, psychotherapy interventions, quality of life with cancer, social support, adoptive coping, end of life, palliative care

## Abstract

Prolonged grief disorder, also known as post-loss grief, was officially recognized in the International Classification of Diseases (ICD-11) after years of debate within the mental health community. However, while post-loss grief gained recognition, anticipatory and preparatory grief, which occur before a loss, have remained underexplored. Preparatory grief affects individuals nearing the end of life, while anticipatory grief impacts the loved ones of those who are about to die. These grief types are particularly prevalent among women, who are more vulnerable to their emotional and psychological challenges. The primary aim of this study was to investigate preparatory grief in women diagnosed with cancer and anticipatory grief in their loved ones, with the goal of developing management guidelines. The secondary objective was to identify protective factors, such as psychotherapeutic interventions and systemic support, to alleviate grief-related distress. This review synthesized evidence from the PubMed and Cochrane databases, covering studies from 1968 to 2020 and after the COVID-19 pandemic in 2023. The results revealed that anticipatory grief was common among loved ones, leading to increased emotional distress, while cancer patients experienced preparatory grief, facing both emotional and practical challenges. Both types of grief were associated with altered stress responses, such as lower diurnal cortisol levels. Psychotherapeutic interventions, particularly early and systemic psychotherapy, were found to effectively reduce symptoms of both anticipatory and preparatory grief, improving coping strategies and emotional well-being. The study concluded that empowering coping strategies and social support played key roles in enhancing emotional outcomes for both patients and their families.

## 1. Introduction

Cancer, a life-altering illness, introduces a wide range of emotional, psychological, and existential losses for both the person diagnosed and those around them. Grief, in this context, is not limited to death; it is tied to the many layers of loss that cancer creates, touching almost every stage of the cancer experience—for patients, caregivers, families, and even medical professionals. Unfortunately, cancer remains a leading cause of death worldwide, with both incidence and mortality rates on the rise [1]. Global cancer statistics 2022: GLOBOCAN estimates of incidence and mortality worldwide for 36 cancers in 185 countries [1]. The Global Burden of Disease 2021 study, conducted by the Global Burden of Disease 2021 Diseases and Injuries Collaborators, presents a detailed analysis of the global, regional, and national impact of 371 different diseases and injuries. The study covers 204 countries and territories, along with 811 subnational regions, over the period from 1990 to 2021. It provides essential information on how often these diseases and injuries occur, how many people are affected, how many years people live with the health consequences of these conditions, how many years of healthy life are lost due to illness or early death, and the overall expectation of living in good health. Including this data in our review allows us to better understand the patterns and trends in the burden of disease around the world and highlights the importance of addressing both fatal and non-fatal health outcomes in global health planning [2]. Based on updated estimates from the International Agency for Research on Cancer (IARC), it is suggested that there were approximately 20 million new cancer cases and 9.7 million cancer-related deaths globally in 2022 [1]. Lung cancer was the most prevalent, accounting for 12.4% of all new cases and 18.7% of all deaths. This was followed by female breast cancer (11.6% of new cases), colorectal cancer (9.6%), prostate cancer (7.3%), and stomach cancer (4.9%) [1]. Projections indicate that the global cancer burden will increase by 77% by 2050, reaching over 35 million annually. This surge will be most pronounced in low- and medium-human development index countries, where cancer-related deaths are expected to nearly double [1]. Screening programs have been shown to reduce cancer incidence and mortality across various types of cancer, including cervical, colon and breast cancer [3,4,5,6]. The goal of cancer screening is to detect cancer early, before there are any symptoms, when it is more likely to be treated successfully and when it can mean simpler treatments and better outcomes. For some, encouraging others to be screened or supporting someone through therapy becomes a way to process and work through grief. In 2012, the Republic of Serbia started organized screening for cervical cancer, colon cancer, and breast cancer, with the Institute of Public Health of Serbia as the responsible institution that carries out organized screening [7,8].

Cancer screening plays a vital role in public health, serving not only in the early detection of cancer but also as a cornerstone of prevention. Timely screening allows for earlier intervention, significantly improving outcomes and survival rates. It also provides a unique opportunity for clinicians to communicate potentially life-changing diagnoses to patients in a way that supports emotional preparedness and clarity [9].

Beyond its clinical purpose, cancer screening serves as the first step in a sensitive and often emotionally charged journey for patients and their families. When a diagnosis of cancer is suspected or confirmed, the way this information is conveyed can profoundly affect both the patient and their loved ones [10]. Communicating the potential for a life-threatening illness initiates what is known as *preparatory grief* in the patient—an emotional process that helps them begin to cope with the possibility of loss. Simultaneously, family members may begin to experience *anticipatory grief*, as they confront the potential future without their loved one [11]. Both anticipatory and preparatory grief highlight the need for a public health approach that supports patients, healthy individuals, and their loved ones through early screening, open communication, and psychological care.

Understanding these forms of grief is essential for all healthcare and public health professionals who may find themselves in the position of being the first to deliver difficult news. Education on preparatory and anticipatory grief equips professionals with the tools to navigate these moments with sensitivity, compassion, and clarity [11,12,13]. It helps prevent unnecessary emotional distress and supports healthier coping processes for patients and families alike. Investing in this knowledge can facilitate communication, improve trust, and ultimately reduce the risk of triggering more complex grieving reactions.

Delivering bad news is an emotionally charged and ethically delicate task that many public health professionals are unprepared for. Understanding and applying concepts such as preparatory and anticipatory grief can enhance the way healthcare providers approach difficult conversations. By integrating this knowledge into public health education and professional training, we not only improve the human side of healthcare but also contribute to better psychological outcomes for patients and families. Supporting professionals through training helps prevent miscommunication, reduces emotional harm, and fosters more compassionate, trust-based patient care [13].

Aggressive types of cancers are considered those most likely to result in death within five years or less after establishing the diagnosis, meaning they form, grow, or spread quickly [14]. Upon receiving such a diagnosis, patients often react with anticipatory grief, in an effort to understand and process future losses and the received information [15]. Moreover, the same kind of reaction can be triggered by receiving a diagnosis of other types of cancer or non-cancer-related but potentially mortal conditions. Anticipatory grief can lead to the experience of negative emotions and decreased quality of life [16]. Women are more susceptible to grief and these negative outcomes. This insight into the female transition from receiving a diagnosis of terminal illness to the end of life or surviving makes it necessary to intervene on time in order to help the patient and improve the quality of life, especially the end-of-life care strategy. In this review, we investigate anticipatory grief in women with cancer and introduce potential interventions to support them and their families or the surrounding environment in this process.

It is important to note that while cancer is indeed a global health concern, with millions of cases diagnosed annually, the key figure in relation to our study is not simply the number of people who develop cancer, nor even the total number of cancer-related deaths. Our focus is not on mortality rates per se, but rather on the grief experiences of the living—those who are confronted with the impending loss of a loved one due to cancer. This includes the anticipatory grief experienced by family members and loved ones as they prepare for the potential death of someone close to them, a process that is distinct from post-loss bereavement.

Our study centers on pre-loss grief and its effects on the loved ones of individuals with terminal cancer. Although cancer mortality statistics are certainly significant, they do not fully capture the emotional and psychological impact of anticipating a loved one’s death. In this context, the grief process begins long before the actual death occurs. This anticipatory grief, particularly among women, plays a critical role in shaping the emotional experiences of individuals coping with the prolonged period of uncertainty, fear, and emotional strain. For these reasons, the primary aim of our review is to explore the psychological and emotional challenges faced by the loved ones of those with terminal cancer, focusing on anticipatory grief rather than cancer mortality rates.

Grief is a common and significant experience for individuals aged 60 and above. The death of a loved one often triggers intense mourning, marked by feelings of longing, a diminished interest in usual activities, and persistent thoughts of the deceased. For the majority, this intense grief gradually transitions into a more balanced state, known as integrated grief, where the individual is able to resume regular activities and rediscover enjoyment or meaning in life. However, approximately 7% of older adults who experience loss will develop complicated grief (CG). In CG, the natural progression from acute grief to integrated grief is disrupted, and the individual continues to experience intense and debilitating grief symptoms that severely impact their daily functioning [17]. The background of prolonged grief disorder becoming officially recognized as a diagnosis and receiving an assigned code in the International Classification of Diseases is a complex and lengthy process. For many years, prolonged grief disorder, also known as post-loss grief, was debated within the mental health community, as experts struggled to define its boundaries and distinguish it from normal grief. After extensive research and discussions, prolonged grief disorder was finally recognized in the International Classification of Diseases, version 11, marking a significant milestone in grief research and solidifying its status as an official diagnosis. However, while post-loss grief has gained recognition, anticipatory and preparatory grief, which occur before a loss, have remained significantly underexplored. Preparatory grief affects individuals who are nearing the end of life, as they process feelings of mortality, while anticipatory grief impacts the loved ones of those who are about to lose someone. These grief types are particularly prevalent among women, who are especially vulnerable to the emotional and psychological challenges they bring. Both forms of grief can profoundly affect emotional well-being and quality of life, creating a need for deeper exploration and intervention. Furthermore, cancer is one of the most prevalent diseases in the world, with 14 million new cancer cases annually [18]. There were an estimated 18.1 million new cases of cancer (all cancers combined, excluding non-melanoma skin cancer) worldwide in 2020: 8.8 million (48%) in females and 9.3 million (52%) in males, giving a male-to-female ratio of 10:9.5 [19]. The world age-standardized incidence rate shows that there are 178.1 new cancer cases for every 100,000 females in the world and 206.9 new cancer cases for every 100,000 males [19]. The four most common types of cancer worldwide are female breast, lung, bowel (including anus) and prostate cancers, which account for more than four in 10 (43%) of all new cases [19]. While the incidence of cancer cases and cancer susceptibility are higher in men [20], the 5-year prevalence is higher in women [18]. Not only do women live longer with a cancer diagnosis, but they are also uniquely affected by two of the most common cancer types: breast and cervical cancer. According to the World Health Organization, these female-related cancers have the first (breast cancer) and fifth (cervical) highest cancer prevalence overall (Breast cancer alone caused 15.4 63 million disability-adjusted life years (DALYs), of which 88% came from years of life lost and 12% from years lived with disability (YLDs) [21]. Gynecological cancers invade female reproductive organs, including the cervix, ovaries, uterus, fallopian tubes, vagina and vulva [22]. While each gynecological cancer has a unique set of symptoms and risk factors, generally, the risk in women increases with age [23]. In the past 40 years, the number of cases of cervical cancer and the number of deaths from cervical cancer have decreased significantly. This decline can mostly be attributed to the regular Papanicolaou tests (PAP smear), which can detect cervical pre-cancer before it turns into cancer. Female patients generally have better cancer-specific survival rates than male patients. After adjusting for cancer prevalence with 1:1 matching, gender remained a significant factor in cancer-specific survival. Among the cancer types included, female patients showed a survival advantage in lung, liver, colorectal, pancreatic, stomach, and esophageal cancers, while male patients had better survival rates in bladder cancer. The gender disparity was consistent across both nonmetastatic and metastatic cancer patients, with the exception of kidney cancer. Overall, gender appears to significantly influence cancer-specific survival, with female patients having better outcomes than male patients in most cancers [24]. To summarize, female patients with a cancer diagnosis live longer and have a higher survival rate. Due to the longer exposure to disease, however, the challenges for psychological coping increase. Anxiety and depression are often comorbid with the expected grieving process that occurs for everyone who faces death [25,26].

In this review, we specifically focus on women aged 40 and above, a group particularly vulnerable to both carcinoma and the psychological challenges of stress, depression, and grieving. As women in this demographic often experience lowered estrogen levels due to perimenopausal or menopausal changes, their susceptibility to heightened stress and complicated grief is significantly exacerbated. This is especially relevant when considering the potential for comorbid conditions, including cancer, as stress plays a key role in their overall health.

Research by Saavedra Pérez et al. (2017) [27] highlights the significant impact of complicated grief on cortisol levels, with altered diurnal cortisol patterns observed in those who experience prolonged or intense grief. The hormonal fluctuations that accompany menopause, combined with the psychological strain of grieving, may worsen the stress response in women over 40 [27]. As Saavedra Pérez et al. (2017) note, the physiological changes tied to both hormonal shifts and grief could contribute to an increased vulnerability to cancer and other stress-related disorders [27]. This makes understanding the intersection of these factors crucial for evaluating the health risks this population faces. Additionally, the role of estrogen receptors in modulating the reactivity of the hypothalamus–pituitary–adrenal axis has been explored in previous studies. Moreover, the administration of 2,3-bis (4-hydroxyphenyl)-propionitrile reduced both adrenal corticosterone and adrenocorticotropic hormone responses to restraint stress, suggesting that estrogen receptors may modulate the hypothalamus–pituitary–adrenal axis and neuroendocrine stress reactivity. Another study demonstrated that estrogen receptor isoforms positively influenced the regulation of corticotropin-releasing hormone promoter activity, further indicating that estrogen receptors are likely involved in the mechanisms of stress responses and the development of disorders related to the hypothalamus–pituitary–adrenal axis [28]. These findings highlight how hormonal factors, including the impact of estrogen on the stress response, may amplify the physiological and psychological challenges faced by women over 40, particularly in the context of cancer and preparatory grief.

**Primary objective:** To investigate preparatory grief in women diagnosed with cancer and anticipatory grief in their loved ones as they confront the impending loss of the cancer patient.

**Secondary objective:** To identify protective factors through psychotherapeutic interventions and systemic support aimed at mitigating grief-related distress

This paper also emphasizes the importance of psychoeducation and family therapy, which can promote better understanding of both types of grief, as the sociological setting does not understand the differences and specificities of this different type of grieving. To ensure a better understanding of the grieving process in daily life, we enclose Table A1. In line with this, we advocate for a multidisciplinary treatment framework that aligns pharmacological and psychotherapeutic interventions to provide holistic, patient-centered care. In Table A1, we present an overview of the effects of psychotherapy on the grieving process and emotional and behavioral pathways.

## 2. Materials and Methods

This comprehensive review was conducted using a structured approach to reviewing the literature. The goal was to synthesize evidence from existing studies on anticipatory and preparatory grief in cancer patients and their families. This review was conducted using a structured approach informed by the PICO framework (population, intervention, comparison, outcomes) and relevant keywords, but it does not meet the criteria of a formal systematic review. While we employed comprehensive databases and applied strict inclusion/exclusion criteria, we did not perform a formal systematic review using Boolean operators, which are typically used to refine the search process. A literature search was performed using two major databases, PubMed and Cochrane Library, to gather relevant studies published between 1968 and 2018. Studies dealing with quantitative data published after 2018 were excluded. Studies dealing with quantitative data published after 2018 were excluded.

This decision was made to avoid potential confounding factors introduced by the Coronavirus Disease of 2019 (COVID-19) pandemic. Given that the pandemic significantly altered global mental health dynamics, the emotional distress and anticipatory grief associated with COVID-19 could overlap with the grief responses of cancer patients and their families. By restricting our review to studies published before 2018, we ensured that the evidence synthesized focused solely on grief related to cancer diagnoses, without the added complexity and bias that the COVID-19 pandemic could introduce.

Including studies from the period of the COVID-19 pandemic could have led to an overestimation of anticipatory and preparatory grief effects. The pandemic likely exacerbated emotional distress across populations, making it difficult to distinguish grief associated specifically with cancer diagnoses from the grief experienced due to pandemic-induced health anxiety and societal instability. By excluding post-2018 studies, we eliminated this confounding variable and ensured that our findings reflect grief experiences attributable to cancer diagnoses alone. At the point of discussion, upon revision, we included the recommended new published research.

A comprehensive search strategy was developed based on the PICO framework (population, intervention, comparison, outcomes). This framework helped define key terms and keywords for the search, focusing on cancer patients, their families, and interventions that address grief-related distress. The population of interest was cancer patients diagnosed with terminal conditions and their loved ones who were undergoing anticipatory grief in preparation for the loss. The intervention of interest included psychotherapeutic treatments and systemic support strategies aimed at alleviating the emotional and psychological burden associated with anticipatory and preparatory grief. Outcomes included emotional well-being, coping strategies, stress levels, and biomarkers, such as cortisol levels.

Inclusion criteria were based on relevance to the study objectives, focusing on studies that provided data on the grief process, grief interventions, and protective factors for cancer patients and their loved ones. Studies that explored the impact of grief on mental and physical health, particularly those that assessed the physiological markers of grief (e.g., cortisol levels), were prioritized. The selected studies were subjected to a detailed quality assessment based on a measurement tool to assess systematic reviews, ensuring that only high-quality, relevant studies were included in the review.

The synthesis of the studies focused on identifying recurring themes related to anticipatory and preparatory grief, gender differences in grief responses, and the efficacy of various psychotherapeutic interventions. Additionally, the review aimed to highlight protective factors that could help mitigate grief-related distress, especially in women, and discuss targeted interventions that could be implemented in clinical settings to improve emotional well-being for both patients and their families. The results were then analyzed to provide a comprehensive overview of the existing evidence and to propose guidelines for managing grief in cancer patients and their families.

## 3. Results

### 3.1. Anticipatory Grief in Loved Ones

#### 3.1.1. Emotional and Psychological Burden

The review identified that anticipatory grief is commonly experienced by the loved ones of cancer patients. In the advanced stages of illness, families are often caught in the dual role of managing complex caregiving responsibilities while simultaneously confronting the emotional reality of an impending loss [29]. The previous study on families of terminally ill cancer patients provided a more precise description of the relational and cultural factors that contribute to family distress [29]. This anticipatory grief commonly manifests as heightened psychological distress, especially among spouses and children, who may become preoccupied with thoughts of death and separation. The emotional burden of anticipatory grief can result in significant anxiety, mood instability, and impairments in daily functioning, such as difficulties with concentration, disrupted sleep, and social withdrawal. Palliative care settings are uniquely positioned to identify families at heightened risk for such distress and to provide tailored support to help them manage the emotional and practical challenges involved. Palliative care professionals themselves often face emotional strain while caring for patients with advanced illnesses, making strong coping strategies essential for their well-being [30]. Early recognition and intervention for anticipatory grief are key to reducing long-term psychological stress and enhancing family resilience. The persistent anticipation of loss contributes to chronic stress, negatively impacting both mental and physical health. Open communication and robust support systems play a vital role in alleviating these effects [29].

#### 3.1.2. Onset and Trajectory of Grief

##### Dimensions and Early Onset of Pre-Death Grief

Grief experienced prior to the death of an individual with a life-limiting illness, often referred to as pre-death grief, is increasingly understood as a multidimensional experience comprising two primary components: anticipatory grief and illness-related grief [31]. Anticipatory grief is oriented toward the future, often expressed through anxiety, emotional distress, and a growing awareness of the looming loss, as family members struggle to imagine life without their loved one physically present [31]. In contrast, illness-related grief reflects a more present-focused sorrow, emerging from ongoing changes, such as the patient’s functional decline, loss of shared roles, and shifts in relational dynamics that occur over the course of the illness [31].

This process can begin early—particularly in cases of terminal diagnosis—when loved ones may start emotionally distancing themselves as a protective or adaptive coping mechanism. Recognizing the onset and dual nature of pre-death grief can help clinicians and support teams better tailor interventions to meet the evolving emotional needs of patients and their families throughout the disease trajectory, especially since previous studies identify the negative psychosocial outcomes associated with the burden of caregiving in conjunction with dysfunctional family relations [32].

This early onset allows loved ones to begin the mourning process before the actual loss occurs. However, it can also lead to post-loss prolonged grief reactions, complicating the adjustment process due to multiple risk factors for developing prolonged grief disorder [33]. The evolution of the prolonged grief disorder diagnosis, including the latest criteria sets for *ICD-11* and *DSM-5*, as well as common comorbid conditions, has only recently been observed, and there is still much to elucidate in the process [33]. Understanding the trajectory of anticipatory grief can aid healthcare providers in offering timely support and interventions. Recognizing the signs early can lead to better coping strategies and emotional preparedness.

#### 3.1.3. Impact on Decision-Making

The emotional burden of anticipatory grief often impairs the ability of caregivers to actively engage in end-of-life discussions and planning. Heightened stress and being psychologically overwhelmed can cloud judgment, delay critical medical and legal decisions, and contribute to a state of decision-making paralysis. These delays may ultimately lead to unmet patient wishes and increase the potential for conflict among family members.

Despite its recognized importance, participation in advance care planning (ACP) remains limited among seriously ill patients, their caregivers, and even healthcare professionals. Multiple barriers—including emotional distress, communication gaps, and uncertainty about prognosis—often hinder timely engagement in this process. Structured and early interventions are needed to support all parties in initiating advance care planning conversations. By providing clear information, emotional support, and practical guidance, healthcare teams can help caregivers make informed choices that align with the patient’s values and preferences, reducing the emotional toll and enhancing the quality of end-of-life care [34].

### 3.2. Preparatory Grief in Cancer Patients

#### 3.2.1. Existential and Emotional Processing

Cancer patients experience preparatory grief as they confront their own mortality. They often express sadness, fear, and anxiety tied to anticipated separation, spiritual uncertainty, and unfulfilled life goals. This grief allows patients to process emotions related to their impending death, potentially leading to a sense of closure. Preparatory grief is a common experience, with emotional distress identified as the strongest independent factor associated with higher levels of grief, regardless of psychological, physical, clinical, or demographic influences [35]. However, unresolved preparatory grief can lead to depression and anxiety. Psychological support during this time is essential to address these complex emotions.

#### 3.2.2. Spiritual and Identity Challenges

Patients frequently re-evaluate meaning, legacy, and spiritual beliefs. Previous studies have demonstrated that most patients faced with life-threatening illness have spiritual needs that are not adequately addressed by their health care providers [36]. Spiritual distress correlates with increased anxiety, whereas existential support improves acceptance. Engaging in spiritual or religious practices can provide comfort and a sense of purpose. Spirituality represents an essential aspect of human life that should be integrated into holistic healthcare. However, a common challenge faced by healthcare professionals in incorporating spirituality into clinical practice stems from the perception of spirituality as a subjective concept, often overlapping with related terms. Consequently, concepts such as spiritual comfort, spiritual care, and spiritual support may benefit from more precise theoretical definitions to guide targeted and effective nursing interventions in the realm of spiritual care [37]. Identity challenges arise as patients cope with changes in their roles and abilities, necessitating support to navigate these shifts. Therapeutic interventions that address spiritual and identity concerns can enhance quality of life in the final stages.

#### 3.2.3. Body Image and Loss of Autonomy

Physical decline and dependency add layers to grief, leading to anticipatory loss of identity and dignity. Changes in appearance and functionality can affect self-esteem and lead to feelings of helplessness. Loss of autonomy is particularly distressing, as it impacts one’s sense of control and independence. Supportive care that promotes autonomy and addresses body image concerns can improve emotional well-being. Encouraging participation in daily activities, as tolerated, can help maintain a sense of normalcy. Targeted therapies, such as dignity therapy, aim to alleviate psychosocial and spiritual distress in the final stages of life. It is unknown yet whether dignity therapy can enhance the sense of dignity and improve psychological and spiritual well-being as well as the quality of life of terminally ill patients [38].

### 3.3. Stress Responses in Grief

#### 3.3.1. Physiological Stress Markers

Both patients and loved ones exhibit low diurnal cortisol levels, a biological indicator of chronic stress [27]. This disruption in the stress system is linked to immune issues, sleep disturbance, and emotional fatigue. Chronic stress can exacerbate existing health conditions and lead to new health challenges [27]. Monitoring stress biomarkers can aid in assessing the need for interventions. Integrating stress reduction techniques, such as mindfulness and relaxation exercises, can be beneficial. Cortisol changes were influenced by factors such as individuals’ emotional responses to grief, depressive symptoms, the intensity of grief, the relationship to the deceased, and demographic factors, such as age and gender. Research on the neuroendocrine aspects of grief is still in its early phases, particularly regarding the measurement of grief and the timing and application of neuroendocrine assessments. While many studies focus primarily on cortisol levels, there is limited exploration of other biomarkers, such as oxytocin. Future studies should aim to investigate a wider array of neuroendocrine markers, using longitudinal approaches to better understand the psychobiological effects of grief. These findings could contribute to the development of personalized psychosocial interventions, especially within palliative care, to help prevent prolonged grief disorder [39].

#### 3.3.2. Trauma-like Symptoms

The intersection of grief and trauma has become an increasingly important focus in recent psychological research, revealing a complex and often overlapping relationship between bereavement and posttraumatic stress. Historically, studies of grief have tended to overlook trauma-related responses, such as those seen in posttraumatic stress disorder (PTSD), focusing instead on emotional pain and depressive symptoms. However, recent evidence suggests that for some individuals—particularly those facing sudden or violent losses—grief may be accompanied by trauma-like symptoms, including emotional numbing, hypervigilance, and intrusive recollections [40]. These reactions, while distinct from typical mourning processes, can significantly disrupt daily functioning and quality of life, pointing to a more complicated psychological response than grief alone. In cases of violent death, research indicates that PTSD symptoms may not only emerge but also persist over time, compounding depressive symptoms and intensifying the grieving process. Notably, it appears that the violent nature of the loss, rather than its suddenness, plays a more critical role in predicting long-term psychological impact [40]. Some individuals show trauma-like symptoms—emotional numbness, hypervigilance, and intrusive thoughts—highlighting the overlap between grief and trauma. These symptoms can interfere with daily functioning and quality of life. Addressing these symptoms requires specialized therapeutic approaches that acknowledge the traumatic nature of anticipatory grief. Trauma-informed care can enhance the effectiveness of interventions. Recognizing these trauma responses within the context of anticipatory and post-loss grief underscores the need for trauma-informed therapeutic approaches. Tailored interventions that acknowledge the dual burden of grief and trauma are essential. Early identification and appropriate psychological care can help prevent these symptoms from evolving into chronic distress or prolonged grief disorder [40]. Early identification and treatment of trauma-related symptoms can prevent long-term psychological issues.

#### 3.3.3. Influencing Factors on Stress Regulation

Stress exerts a profound influence on emotional well-being, behavior, and overall health [41]. While short-term stress responses can be adaptive—particularly in younger or physically healthy individuals—chronic or repeated exposure to stressors may have detrimental health effects, especially among older or vulnerable populations [41]. The physiological toll of stress is shaped not only by the intensity and duration of the stressor but also by personal factors, such as genetic predisposition, existing health conditions, and prior psychological experiences [41]. Importantly, individuals with stronger psychosocial resources—such as emotional resilience, supportive social networks, and effective coping strategies—tend to experience lower biological stress responses.

These protective factors can reduce the risk of stress-related illness and improve health outcomes over time. Consequently, psychosocial interventions are increasingly recognized as essential tools in both mitigating stress and influencing the trajectory of chronic conditions. Moreover, psychosocial interventions have proven useful for treating stress-related disorders and may influence the course of chronic diseases [41].

Support networks, resilience, and mental health history significantly impact biological stress responses. Individuals with strong social support and coping skills may experience less physiological stress. Conversely, those with a history of mental health issues may be more susceptible to stress-related health problems. Also, robust evidence suggests that chronic stress plays a significant role in the onset of severe and impairing psychiatric conditions, including major depressive disorder, bipolar disorder, and posttraumatic stress disorder [42].

Personal factors, such as optimism and adaptability, can influence stress responses. Individual characteristics—such as optimism, adaptability, and a positive outlook—can significantly shape how a person responds to stress, either buffering its impact or intensifying its effects, depending on their resilience and coping style [43]. Stress is a critical factor influencing mood, behavior, well-being, and overall health. While short-term stress responses in healthy individuals may be adaptive, chronic or unrelenting stress—especially in older or medically vulnerable populations—can lead to harmful health consequences. The impact of stress on health is shaped not only by the nature, duration, and intensity of the stressors but also by individual vulnerability, psychosocial resources, and coping mechanisms. For instance, individuals with strong support systems and higher resilience often exhibit more regulated physiological stress responses, potentially protecting them from long-term health deterioration. Among the key personal traits affecting how people manage stress are optimism and adaptability. These psychological characteristics can modulate emotional and biological stress responses, influencing how well individuals adapt to loss, trauma, or chronic caregiving demands. Those who possess a greater capacity to reframe adversity, maintain positive expectations, and adjust to changing realities tend to experience lower levels of stress-related biomarkers and improved emotional outcomes [43]. Moreover, the interplay between personal history—such as prior mental health challenges—and current coping capacity plays a significant role in stress regulation. Individuals with histories of anxiety or depression may be more susceptible to heightened stress reactivity, while those who have developed constructive coping patterns may fare better under pressure. These findings emphasize the importance of integrating psychosocial assessments into healthcare, particularly in palliative and bereavement care settings, where individuals often face cumulative emotional stressors. Targeted psychosocial interventions that enhance resilience and coping skills have shown promise in reducing stress-related symptoms and may positively influence the trajectory of chronic illnesses or grief. By identifying and supporting individual psychological strengths, healthcare providers can tailor interventions that buffer the negative impact of prolonged stress, potentially improving quality of life and long-term health outcomes. Tailoring interventions to individual needs can enhance their effectiveness.

### 3.4. Effectiveness of Psychotherapeutic Interventions

#### 3.4.1. Types of Effective Therapies

Psychotherapeutic interventions are known to enhance emotional well-being in both patients and their families, particularly in the context of chronic and emotionally taxing health conditions. Approaches such as cognitive–behavioral therapy, meaning-centered therapy, and mindfulness have demonstrated effectiveness in managing grief-related distress. Cognitive–behavioral therapy helps individuals to eliminate avoidant and safety-seeking behaviors that prevent self-correction of faulty beliefs, thereby facilitating stress management to reduce stress-related disorders and enhance mental health [44]. This therapy enables individuals to reframe maladaptive thoughts, while meaning-centered therapy helps cultivate a sense of purpose. Furthermore, mindfulness techniques encourage present-moment awareness and emotional regulation. Mindfulness-based psychotherapy is a therapeutic approach that integrates mindfulness principles—such as present-moment awareness, emotional acceptance, and non-judgmental observation—into conventional psychotherapeutic frameworks. This method has gained increasing recognition for its ability to address a wide range of psychological and physical conditions, including depression, anxiety, chronic stress, trauma, and grief. It supports clients in developing greater self-awareness, emotional regulation, and psychological flexibility, which can be particularly beneficial in contexts involving uncertainty, loss, or chronic illness [44]. Mindfulness-based interventions (MBIs), such as mindfulness-based cognitive therapy and acceptance and commitment therapy, have shown promising outcomes in both clinical and community settings [44]. These approaches are generally considered safe, cost effective, and adaptable across diverse populations. However, further empirical research is needed to explore their long-term effectiveness, mechanisms of change, and application in underrepresented groups. Despite these gaps, mindfulness-based psychotherapy continues to evolve as a holistic and compassionate modality, bridging mind-body practices with evidence-based mental health care. Personalized therapy plans that integrate these modalities tend to produce the most favorable outcomes. This therapeutic efficacy is especially relevant for individuals with severe mental illnesses (SMI), such as schizophrenia, major depressive disorder, and bipolar disorder—conditions that are chronic, debilitating, and carry substantial personal, familial, and societal costs. Research has shown that the emotional climate within families, including levels of support, criticism, or emotional overinvolvement, plays a crucial role in either mitigating or exacerbating psychiatric symptoms and relapse risks [45]. Family-based psychosocial interventions have emerged as a valuable strategy for improving clinical outcomes in SMI populations. These interventions often combine psychoeducation with skill building to strengthen family dynamics, foster supportive communication, and reduce relapse rates [45]. However, despite their benefits, systemic barriers—including financial limitations, limited access to specialized care, stigma, and social marginalization—frequently hinder families from initiating or sustaining these services [45]. To ensure wider access and long-term engagement in family-based therapies, there is a need for policy-level initiatives that support integrated healthcare systems, fund community mental health resources, and collaborate with national organizations to reduce stigma and facilitate access to care. When integrated into holistic treatment models, these supports can significantly enhance the impact of psychotherapeutic interventions for patients with SMI and their families alike.

#### 3.4.2. Family-Based and Narrative Approaches

Families are generally unaware and lack information about mental illnesses and how to deal with them, and in turn, may end up maintaining or perpetuating the illness [45]. Family therapy facilitates communication and meaning making. Narrative and legacy-focused sessions helped individuals prepare emotionally for loss. Narrative approaches, such as dignity therapy and life review sessions, encourage patients to reflect on and share their life stories, values, and achievements [38]. This can provide a profound sense of closure and help families preserve emotional connections even after death. Family-centered interventions also promote open conversations about difficult topics, such as end-of-life wishes and emotional preparedness, which can reduce conflict and misunderstandings. Previous studies have pointed towards the importance of family in end-of-life communication and illustrated that family participation likely leads to improved quality of life and death in patients [46]. Moreover, involving multiple family members allows for a shared grief process, where individuals can validate each other’s emotions and collaborate on care decisions. These therapeutic spaces can be critical for healing, especially in emotionally charged terminal care settings.

#### 3.4.3. Timing and Impact

Early interventions—ideally at diagnosis—proved more effective than those introduced later. Proactive psychological support is critical. Proactive psychological programs are increasingly recognized as essential tools for managing occupational stress among individuals in high-demand and emotionally intensive professions. Rather than waiting for symptoms to emerge, these programs aim to strengthen emotional resilience, improve coping strategies, and enhance overall mental well-being before stress becomes overwhelming. Through techniques such as mindfulness training, cognitive–behavioral strategies, peer support, and psychoeducation, workers are equipped to better navigate the psychological demands of their roles [47]. These initiatives are particularly beneficial in fields such as healthcare, emergency response, education, and social services, where sustained stress exposure can lead to burnout and reduced performance. By fostering early awareness and promoting a culture of mental health support, proactive stress management programs contribute to healthier work environments, improved team functioning, and long-term psychological sustainability [47]. Engaging in psychological support early allows patients and caregivers to build emotional resilience before the most intense phases of illness begin. Previously published evidence shows that interventions started shortly after diagnosis are linked with better long-term outcomes, including improved emotional regulation, greater acceptance, and reduced symptoms of depression and anxiety. Delayed support, on the other hand, may be less effective, as patients and families are often too overwhelmed or physically compromised to engage deeply in therapy. Postponing intervention for individuals experiencing acute mental health crises can result in a range of adverse outcomes. When timely treatment is not provided, the situation often escalates, leading to greater reliance on involuntary or coercive care measures, increased physical health complications, higher long-term healthcare expenses, and a heightened risk of the illness becoming resistant to treatment. In cases where systemic or legal obstacles—such as extended delays in securing a Rogers treatment order—prevent appropriate care, patients frequently endure repeated psychiatric crises. Without timely stabilization, conditions such as untreated psychosis or mania can rapidly deteriorate, causing ongoing disruptions that affect both the individual and the healthcare system more broadly [48]. This evidence strongly supports integrating mental health services into routine cancer care, not as a reactive measure but as a foundational component of supportive oncology.

### 3.5. Role of Social Support and Coping Strategies

#### 3.5.1. Community and Peer Support

Peer support groups, both in-person and online, were highlighted as protective resources. In addition, there is little dissemination of knowledge regarding the types and diversity of online grief support [49]. These groups offered not only emotional validation but also practical advice for managing caregiving stress and preparing for loss. Being part of a peer group allows individuals to share experiences and coping mechanisms in a safe, understanding environment. These groups often help normalize emotions, such as guilt, anger, or despair, which can otherwise feel isolating. Structured support programs facilitated by trained counselors further empower participants with communication skills, stress management strategies, and grief education. Online communities provide flexibility and accessibility, particularly for caregivers who may be homebound or juggling multiple responsibilities. Research consistently finds that individuals who participate in support groups demonstrate greater psychological resilience, better coping, and lower levels of complicated grief symptoms post-loss [49].

#### 3.5.2. Coping Styles and Gender Variance

Women preferred emotional and expressive coping; men leaned toward task-oriented strategies. Tailored support plans are essential. Coping mechanisms vary significantly between genders, often shaped by social norms and cultural expectations [50]. The observed sex differences in the use of coping strategies and their association with depression and anxiety-related problems underscore differences in the clinical presentation of anxiety and depression between women and men [51]. Women are more likely to seek emotional support, engage in open grieving, and benefit from expressive therapies, such as journaling or group discussions. Men, on the other hand, often suppress emotions, instead focusing on tasks or logistical problem-solving, such as arranging medical appointments or financial planning. Furthermore, men expressed prolonged grief as an acute, decreasing reaction, whereas women showed an adjourned, mounting grief reaction [50]. These patterns suggest the need for differentiated intervention strategies. For example, therapy for men might emphasize active planning, practical resources, and structured goals, while interventions for women may focus more on emotional expression and relational healing.

#### 3.5.3. Cultural Norms and Caregiving Role Expectations

Cultural norms and caregiving roles intensified grief for women, often leading to emotional and physical exhaustion. Across diverse cultural contexts, women consistently assume the primary role in providing informal care for family members living with chronic illnesses, disabilities, or mental health conditions [52]. These caregiving responsibilities are often shaped by deeply rooted societal norms and gendered expectations that position women as natural caregivers. As a result, they are more frequently exposed to caregiving-related stressors and may experience, interpret, and manage these challenges differently from men, as suggested by stress and coping theories. In many settings, this disproportionate caregiving burden intersects with personal grief, compounding emotional strain [52]. Women caring for seriously ill or aging relatives often face chronic stress, identity conflicts, and a sense of emotional invisibility, as their own psychological needs may be minimized or neglected. The cumulative impact of these demands can intensify feelings of isolation and hinder healthy grief processing. Additionally, some cultural frameworks discourage emotional expression, which may prevent women from seeking help. Women may prioritize the emotional needs of others above their own, suppressing their grief and leading to longer-term mental health consequences. Recognizing these socio–cultural influences is essential in designing effective, culturally competent interventions that acknowledge role-based pressures and offer appropriate support [52].

### 3.6. Gender Differences in Grief Response

#### Emotional Expression vs. Avoidance

Women displayed higher emotional intensity and benefited from expressive interventions. Men exhibited avoidant or stoic grief styles, favoring solution-focused strategies. Emotional expression, while healing for many women, can also be a double-edged sword if not met with adequate social or therapeutic support. When women’s emotional openness is invalidated or misunderstood, it may contribute to feelings of shame or alienation. With respect to gender differences in coping styles, women are more confrontive and expressive of their emotions than men, but there has been little validation of the generally accepted grief work hypothesis that working through grief by women brings about their better recovery [53]. Men, meanwhile, may resist acknowledging grief altogether, often misinterpreted as disinterest or emotional coldness [53]. However, this suppression can lead to cumulative stress, increased alcohol or substance use, and relational strain. Psychosocial interventions must, therefore, normalize diverse symptoms within the grieving process.

In summary, gender differences were clearly observed, with women being found to be more vulnerable to both anticipatory and preparatory grief. This result suggests that women may require more targeted psychological interventions and support to address their unique emotional needs and better manage the distress associated with cancer diagnoses and loss.

Women are generally more emotionally expressive than men, which can intensify their experience of grief. They are more likely to acknowledge and verbalize their emotions rather than suppress them, and often seek emotional support from friends and family—behaviors that can reinforce and deepen the grieving process. Additionally, women tend to ruminate more on their emotions, a cognitive style associated with prolonged feelings of sadness and distress. This openness to emotional experience can make grief feel more immediate and intense, in contrast to men, who may be more inclined to cope through emotional suppression or problem-solving strategies [54].

Biological factors also contribute to these differences. Hormonal variations between men and women influence the intensity of preparatory grief. Estrogen, for example, is linked to heightened emotional sensitivity and stronger stress responses, making women more prone to intense emotional reactions. Moreover, women are more likely than men to experience anxiety and depression, conditions that can amplify the emotional impact of anticipated loss. These physiological and psychological differences suggest that women may be more vulnerable to the emotional weight of anticipatory grief [55].

While both men and women experience preparatory grief, women may be more affected due to their emotional expressiveness, caregiving roles, hormonal influences, social expectations, and strong empathetic connections [56]. These factors make grief a more immediate and profound experience for many women. However, it is important to recognize that grief is deeply personal, and individual coping mechanisms play a significant role in how one experiences loss.

Grief can also create emotional barriers to cancer screening, including fear of diagnosis (especially if cancer feels inevitable after a loved one’s death), avoidance of medical settings, which may trigger memories or trauma, depression, or apathy, leading to neglect of personal health. In these cases, grief can delay or prevent necessary screening, increasing long-term risk [57].

## 4. Discussion

### 4.1. The Multifaceted Nature of Grief in the Cancer Journey

Cancer is not solely a medical condition—it is a life-altering event that induces complex emotional and psychological responses. The experience of grief is not reserved for end-of-life scenarios but often begins with the diagnosis itself. Patients grieve the loss of health, certainty, and identity, while families and caregivers mourn the imagined future that may no longer be possible. Even healthcare professionals are not immune, as repeated exposure to such emotionally intense scenarios may leave lasting psychological impacts. Recognizing this spectrum of grief is essential in comprehending the broader psychosocial impact of cancer [58,59].

### 4.2. The Growing Burden of Cancer: A Global and National Perspective

Cancer’s continuing rise in both how often it occurs and how many people it kills, especially in countries with low and medium levels of human development, highlights an urgent challenge for public health. The worldwide data from the year 2022—20 million new cases and 9.7 million deaths—serve as a serious reminder that progress is not happening equally across all regions. With future estimates predicting a 77 percent increase in the number of cancer cases by the year 2050, healthcare systems around the world will be under growing pressure to find cancer earlier, provide better treatment, and offer stronger support for patients throughout their care. In Serbia, as elsewhere, national health systems must adapt to these realities with proactive, well-coordinated screening strategies [60,61].

### 4.3. Screening as a Clinical and Emotional Intervention

Organized cancer screening programs, such as those introduced in Serbia in 2012 for cervical, colon, and breast cancer, not only reduce mortality but also represent the beginning of a deeply personal journey. Early detection means improved prognosis, yet it also involves disclosing potentially life-threatening information. Here, screening intersects with grief: patients may be plunged into preparatory grief upon diagnosis, while family members begin to process anticipatory grief. This phase, though medically early, is emotionally intense, highlighting the need for integrated communication strategies within public health practice [62].

### 4.4. The Role of Education in Managing Grief Communication

Delivering difficult news, particularly a cancer diagnosis, is one of the most emotionally challenging tasks that healthcare professionals face. Unfortunately, many public health practitioners lack formal training in how to approach these sensitive moments, leaving them ill-prepared to manage the emotional complexities that arise. As a result, the conversation about a life-altering diagnosis can inadvertently exacerbate the psychological burden on patients and their families [12].

Education on how to handle preparatory and anticipatory grief should be an integral component of healthcare training. Understanding these concepts equips professionals with the skills to deliver bad news in a manner that is both compassionate and clear, minimizing potential emotional distress. Additionally, a well-informed approach can help healthcare providers identify when a patient or family member is experiencing grief and initiate appropriate support measures, thereby improving the emotional experience of the patient and their loved ones [12,63].

Training that includes specific guidance on grief communication could also improve interprofessional collaboration. Doctors, nurses, counselors, and public health practitioners who are equipped with similar training will communicate more cohesively, leading to more effective patient-centered care. This unified approach reduces the likelihood of miscommunication and ensures that the entire healthcare team is attuned to the emotional needs of the patient and family, fostering a supportive, trusting environment [13,64].

Furthermore, education on grief management helps to normalize the emotional responses that accompany life-threatening illnesses. Rather than viewing grief as an isolated or negative reaction, healthcare professionals who are trained in anticipatory and preparatory grief can understand these emotional responses as part of the healing process. By doing so, they not only improve the quality of care but also create an environment where patients and families feel heard, understood, and supported during one of the most difficult times of their lives [12,13,63].

### 4.5. Understanding the Psychological Impact of Cancer on Women: Coping, Distress, and Adaptation

**Figure A1:**
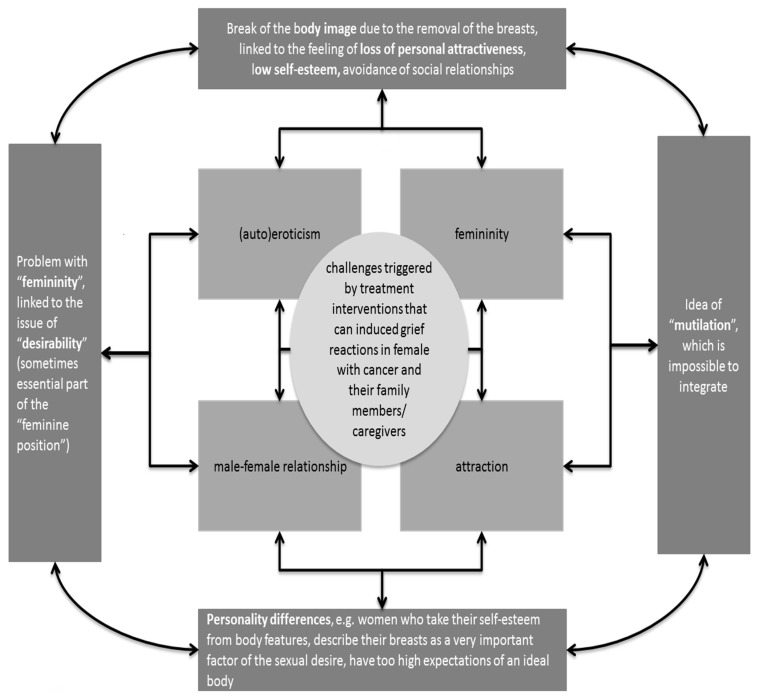
Psychological impact triggers in women following cancer surgery [78].

Coping with a cancer diagnosis is challenging, as it demands significant psychological adjustment from patients. Coping involves both actions and thoughts to manage stressors threatening one’s psychological well-being [65]. A cancer diagnosis presents a specific, substantial challenge, leading to negative health outcomes and requiring patients to confront complex coping difficulties [66,67,68,69]. These challenges can be personal, such as facing a threat to life or dealing with disrupted life functions [70,71], or interpersonal, such as the potential separation from loved ones [70,71,72]. Additionally, patients must navigate decisions about future treatments [70], and family members may experience anticipatory grief, marked by increased concern for the dying person, visualizing the death, and preparing for life after the loss [73]. Women often face unique distress upon receiving a cancer diagnosis. A study was conducted to explore how women aged 40 and above, who were about to undergo a breast biopsy, responded to various emotional and psychological factors, including their personality, cognitive appraisal, coping strategies, and mood. The women were assessed before the biopsy, after receiving their diagnosis, and for those who were diagnosed with cancer, after their surgery. Upon biopsy, 36 women received a cancer diagnosis, while 81 were informed their results were benign. Initially, both groups showed no significant differences in their appraisals, coping methods, or emotional states before the diagnosis. However, after the biopsy, those diagnosed with cancer reported higher levels of negative emotions compared to those with benign results. Post-surgery, women with cancer experienced more fatigue and less vigor than those in the benign group, although the two groups showed no significant differences in other negative emotions [74]. These findings indicate that psychotherapy treatment could play a crucial role in addressing the long-term psychological well-being of women diagnosed with cancer. The other study revealed that women who were depressed at the time of treatment planning and who reacted to their cancer diagnosis with cognitive avoidance, such as acceptance or resignation, experienced significantly worse psychological adjustment three years later. This poor adjustment was strongly linked to cognitive avoidance and a limited use of approach-based coping strategies. These findings highlight that women who respond passively to their breast cancer diagnosis, through resignation or avoidance, are at a heightened risk for ongoing psychological distress. Therefore, psychological interventions for these women should focus on addressing cognitive avoidance, promoting active coping mechanisms, and enhancing overall well-being [75]. In addition to the aforementioned common fears, such as the fear of dying or cancer recurrence, women with female-related cancers may experience distress related to changes in self-image, femininity, and sexual attractiveness, especially after procedures, such as mastectomy, hysterectomy, or trachelectomy [76,77,78]. The potential loss of fertility due to chemotherapy or radiotherapy can further compound emotional challenges, particularly for women aged 15–49 [79]. The grief underscores the necessity for a public health approach that includes screening followed with compassionate communication, and psychological support for healthy individuals, family members, and loved ones, as well as those with diminished health [80]. Figure A1 summarizes the key aspects triggering psychological issues in women with cancer [78]. Bereavement, the process of experiencing the loss of a loved one, can accompany cancer diagnoses, often leading to grief [81]. Grief involves a combination of thoughts, emotions, behaviors, and physiological changes, varying in intensity and expression over time [81]. Mourning, a related process, refers to integrating the finality of the loss into memory and ultimately reintroducing joy into life [81]. Table A1 provides an overview of key terms used in relation to grief and sadness. Approximately 15% of grievers experience complicated grief, marked by excessive ruminations, avoidance, or difficulty regulating emotions [81,82,83]. Complicated grief is distinguished from uncomplicated grief by its persistence and intensity, leading to greater distress and disability [84]. The Inventory of Complicated Grief is the most commonly used scale to assess grief severity [85]. It is known that some of the relevant factors for grief predisposition are female gender, presence of higher depressive symptoms, lower education, and difficulties in daily activities (which might be, in the sense of the current discussion, viewed as a consequence of the cancer diagnosis) were independently associated with a higher bereavement severity [86]. Furthermore, people with any form (post-loss or pre-loss (anticipatory or preparatory)) are expected to have low levels of morning cortisol and low overall diurnal cortisol levels characteristic of a chronic stress reaction [27]. For an in-depth discussion on complicated grief, see Boelen and Smid [82]. Anticipatory grief refers to emotional responses to an impending loss, allowing individuals to prepare for the loss of a loved one or themselves, especially in terminal illness [87,88]. Women, particularly, experience heightened anticipatory grief, which may stem from their greater expressiveness of emotions [72]. Early interventions targeting anticipatory grief can reduce both the severity of symptoms and post-mortem grief in loved ones [89]. Anticipatory grief differs from preparatory grief, which involves practical preparations for an impending loss, such as arranging personal affairs and making logistical decisions [90]. A broad range of determinants influencing patient adherence to treatment regimens is identified, many of which are rooted in the dynamics of the doctor-patient interaction. Nonadherence can occur when a practitioner fails to effectively communicate aspects of the treatment, such as not discussing the regimen, offering inconsistent instructions, using technical language that patients cannot understand, or lacking rapport with the patient. Additionally, failing to monitor the patient’s adherence during follow-up visits can contribute to nonadherence. These factors are closely aligned with the guidelines outlined in Table A2: Guidelines for Communication with Cancer Patients and Their Loved Ones. Details are presented in the table [91,92]. Research has found that negative illness representations are linked to increased grief symptoms [93]. Healthcare providers can mitigate the negative impact of these representations by offering clear, informative communication, improving patients’ understanding of their condition and promoting better coping strategies [94]. Social networks play a critical role in supporting cancer patients. Family, friends, and healthcare professionals offer emotional and practical support, which positively affects patients’ well-being [95]. However, caregivers also experience anticipatory grief, and supporting them through psychotherapy can improve both their coping and the family’s overall cohesion [96]. A narrative approach, encouraging caregivers to express their emotions and redefine their roles, has shown promise in enhancing coping [97]. Effective interventions, including psychotherapy and social support, are vital for cancer patients and their families. Early intervention can help prevent severe grief, depression, and anxiety in female patients. Furthermore, recognizing gender-specific challenges, such as fertility and sexual health concerns, allows for tailored support [79]. Psycho-oncological care addresses both emotional and practical challenges of the patient’s health [98]. The International Psycho-Oncology Society’s mission is to promote global excellence in the psychosocial care of people affected by cancer through research, public policy, advocacy, and education. The goal of society is to improve the quality of life for cancer patients and their families by integrating psychosocial care into cancer treatment. This approach ensures that emotional, mental, and social well-being are addressed alongside physical health, acknowledging the significant impact psychological support has on treatment outcomes and overall quality of life. The society advocates for this integrated care model to provide comprehensive support throughout the cancer journey. Society’s standard of quality cancer care clearly states the importance of incorporating the psychosocial domain into routine care [99]. We present the details regarding psychotherapy interventions for preparatory and anticipatory grieving in Table A3, including how it helps the individual with cancer, the patient’s loved ones, clinical healing, and clinicians.

In summary, cancer diagnoses significantly affect both patients and their families, especially women. Early psychological interventions, focused on anticipatory grief, social support, and effective coping strategies, can improve emotional well-being and quality of life. Future research should focus on identifying protective factors and refining interventions to support women facing a cancer diagnosis and their loved ones, and better identify the difference between preparatory and anticipatory grief to target the interventions better. In Table A4, we highlight the key distinctions between anticipatory and preparatory grief, and how illness representations (i.e., how individuals perceive and understand their illness) can significantly influence coping strategies during the grief process.

### 4.6. Unseen Grief: What Clinicians Are Missing in Addressing Anticipatory and Preparatory Grief in Cancer Care

Clinicians often overlook the psychological complexities of anticipatory and preparatory grief in cancer patients and their loved ones, focusing primarily on physical symptoms and medical treatments. While cancer care is predominantly driven by clinical protocols, the emotional and psychological well-being of patients, especially in the context of terminal diagnoses, remains underemphasized. Anticipatory grief, experienced by loved ones, is marked by emotional distress as they prepare for the inevitable loss, but this form of grief is often misunderstood or dismissed as a natural consequence of cancer caregiving. Similarly, preparatory grief, which cancer patients themselves experience while preparing for their death, can manifest as emotional and existential challenges, yet it is frequently not acknowledged by clinicians. Many healthcare providers fail to recognize that patients and their families can experience profound grief long before the actual loss occurs. This gap in understanding can lead to inadequate emotional support, leaving patients and their families to cope with complex feelings of fear, sadness, and hopelessness on their own.

To address this gap, clinicians should integrate psychological interventions, such as short-term psychotherapy, into their care plans. Cognitive–behavioral therapy (CBT) can be particularly effective, helping both patients and their families reframe their emotions and develop coping strategies to manage anticipatory and preparatory grief. Additionally, narrative therapy can provide patients and their loved ones with a space to express their emotions and reframe their story in a way that acknowledges their grief while also fostering resilience. Mindfulness-based interventions can help reduce the emotional distress associated with both types of grief, allowing individuals to remain present and cope with overwhelming feelings of loss. Family therapy can also be critical, offering a supportive environment for caregivers and loved ones to share their grief and gain a better understanding of the grieving process. The failure to address anticipatory and preparatory grief, coupled with an absence of timely, patient-centered psychological interventions, can contribute to worsening mental health outcomes, such as depression and anxiety. A more comprehensive approach that incorporates grief counseling, emotional support, and coping strategies into the overall treatment plan is essential. Clinicians need to recognize that the emotional journey through cancer extends far beyond physical health and requires dedicated attention to the psychological aspects of the patient and their family’s experience.

### 4.7. How to Integrate Psychotherapy in Terminally Ill Cancer Patients and Their Families Every Day

Integrating psychotherapy into standard terminal cancer care is an important step in providing holistic support to patients. Psychotherapy can help patients manage the emotional, psychological, and existential challenges associated with terminal illness [100]. The following are some ways clinicians can integrate psychotherapy into care.

Integrating psychotherapy into terminal cancer care should begin early to address emotional and psychological challenges before they become overwhelming [101]. Clinicians should conduct early assessments of a patient’s emotional and mental health to identify any signs of anxiety, depression, or anticipatory grief. Recognizing these concerns early can help ensure that appropriate support is provided in a timely manner. Additionally, regular screenings for mental health issues, such as the Hospital Anxiety and Depression Scale (HADS) or the Distress Thermometer, should be routinely implemented [102]. These screenings allow clinicians to identify patients who may benefit from psychotherapy, ensuring that mental health is consistently monitored and addressed throughout the course of care.

A collaborative, interdisciplinary approach is essential in integrating psychotherapy into cancer care [103]. Oncologists, nurses, palliative care specialists, and psychotherapists should work together to provide coordinated care that addresses both the physical and emotional needs of the patient. This teamwork ensures that psychological support is seamlessly integrated into the patient’s treatment plan. Oncologists, in particular, can play a key role by closely monitoring their patients for signs of psychological distress and referring them to clinical psychologists or counselors for psychotherapy sessions when needed. This referral process helps ensure that patients receive the emotional support necessary to navigate the challenges of terminal illness.

Psychoeducation plays a crucial role in preparing both patients and their families for the emotional journey of terminal cancer [104]. Offering discussions about anticipatory grief, fear of death, and acceptance can help individuals better understand and navigate the complex emotional responses they may encounter. Additionally, teaching coping strategies, such as mindfulness, relaxation techniques, and cognitive behavioral strategies, can provide patients with effective tools to manage anxiety, pain, and emotional stress. By equipping patients and their families with these resources, clinicians can help foster emotional resilience and promote better overall psychological well-being throughout the course of the illness.

Tailoring psychotherapy to the individual needs of cancer patients is essential for providing personalized emotional support [105]. Individual therapy, such as cognitive–behavioral therapy (CBT), can be highly effective in helping patients cope with negative thought patterns, addressing issues like anxiety and depression, and fostering emotional resilience. Additionally, existential or meaning-centered therapy (MCT) can help patients explore life’s meaning, dignity, and existential concerns, providing them with an opportunity to find peace and acceptance in their final days. For many patients, involving family members in therapy sessions can also be beneficial. Family therapy helps facilitate open communication, resolve conflicts, and ensures that the patient’s emotional needs are met within the family context, promoting a supportive and compassionate environment during a challenging time.

Offering group therapy or support groups for cancer patients can provide a sense of community and reduce isolation. Peer support allows patients to share their experiences, validate each other’s feelings, and gain strength from others in similar situations [106].

Psychotherapy can also be directed toward caregivers, who may experience significant stress, burnout, and grief. Providing them with coping strategies can improve their well-being and enhance their ability to support the patient [107].

Teach family members how to offer emotional support to their loved ones in terminal stages, encouraging open communication and preparing them for the grieving process [107].

A diagnosis of cancer often brings up existential concerns about death and legacy. Psychotherapy can guide patients in addressing fear of death, achieving closure, and finding meaning in their life story.

Psychotherapists can help patients develop emotional resilience, cultivate acceptance, find peace, and foster emotional readiness for the end-of-life process [108].

Offering psychotherapy through telehealth platforms can be especially helpful for patients who are physically unable to visit a therapist or live in remote areas. Introducing patients to online resources, mindfulness apps, or virtual support groups may complement in-person therapy [109].

### 4.8. Future Research Directions: Early Intervention and Psychotherapy Efficacy in Preparatory and Anticipatory Grief

Another key area for future research is the exploration of early psychotherapeutic interventions for both cancer patients (preparatory grief) and their loved ones (anticipatory grief). This review suggests that early systemic psychotherapy and other support mechanisms significantly alleviate grief in both contexts. Future studies should focus on refining intervention models, assessing the most effective timing and formats for therapy, and understanding how different psychotherapeutic techniques—such as cognitive behavioral therapy or narrative therapy—may specifically impact grief processing in both preparatory and anticipatory grief. Furthermore, examining how early psychological support affects long-term coping abilities and the overall quality of life is crucial.

### 4.9. Protective Factors and Systemic Support in Preparatory and Anticipatory Grief

The review also points to the importance of identifying protective factors that may mitigate grief-related distress in both preparatory and anticipatory grief. Further research should investigate the role of social support networks, family dynamics, and community involvement in buffering the emotional impacts of both types of grief. A focus on systemic approaches, which integrate family members and close social networks into therapeutic interventions, could provide valuable insights into how these external supports influence grief outcomes. Researchers may also examine the feasibility and effectiveness of digital interventions or online support groups as part of systemic support systems for both cancer patients and their families.

### 4.10. Predicting Long-Term Psychological Outcomes in Preparatory and Anticipatory Grief

Lastly, there is a critical need for studies focused on predicting long-term psychological outcomes for cancer patients (preparatory grief) and their families (anticipatory grief) dealing with these grief processes. While short-term interventions have shown promise, it is important to understand how these interventions translate into lasting improvements in mental health and quality of life. Longitudinal studies that track grief trajectories over time will help refine predictions about who may be at higher risk for chronic distress, allowing for more targeted interventions to be developed for both types of grief.

### 4.11. Healthcare System Implications and Public Health Policy for Supporting Women with Cancer and Their Loved Ones Through Targeted Psychotherapy and Other Interventions Against Preparatory and Anticipatory Grief

Public health and bereavement support may be seen through programs aimed at increasing cancer screening, which often target those affected by cancer loss, especially in at-risk populations; support groups may include health education components to encourage preventive care. Some grief-centered campaigns (like Breast Cancer Awareness Month) promote both remembrance and prevention [110].

The psychological impact of both preparatory and anticipatory grief on women with cancer and their loved ones is profound, often leading to emotional distress and reduced quality of life. This review highlights the need for targeted psychotherapeutic interventions and systemic support to address these challenges. The healthcare system must integrate mental health care into cancer treatment, offering specialized grief counseling for patients and their families from the early stages of the diagnosis. Mental health professionals, including therapists trained in grief and palliative care, should be part of oncology and palliative care teams to provide timely and effective support.

Public health policies should emphasize the importance of early intervention for both preparatory and anticipatory grief. Policy changes can advocate for routine grief support as part of cancer care, ensuring that women facing terminal diagnoses and their loved ones have access to the resources they need. Furthermore, the development of community-based interventions, such as peer support groups or digital platforms for remote therapy, could provide additional layers of support, especially for women who may experience grief more intensely.

In addition, healthcare policies should incorporate gender-sensitive approaches, recognizing that women may experience both anticipatory and preparatory grief differently. Targeted interventions that address these gender differences in emotional processing could enhance coping mechanisms and help reduce prolonged distress. By implementing these psychotherapeutic strategies and policies, healthcare systems can better address the needs of women and their families, improving emotional well-being and psychological resilience during a challenging time.

As highlighted in this review, women appear more vulnerable to grief-related distress, with heightened emotional reactions and potentially greater long-term psychological impacts in both types of grief. Future studies should explore the biological, social, and cultural factors that contribute to these gendered differences. It will be important to identify whether gender-specific interventions could be more effective in addressing grief in women compared to men, and whether different types of psychotherapeutic approaches should be tailored accordingly to enhance emotional well-being in women diagnosed with cancer and their families.

Likewise, grief can be a catalyst for preventive action. After losing someone to cancer, individuals often become more aware of their own health risks, especially if there’s a hereditary component. Grief, in this context, can become a powerful motivator for seeking early detection through screenings (e.g., mammograms, colonoscopies, genetic testing), changing health behaviors, such as diet, smoking, or exercise, and encouraging others in the family to be screened.

Grief, as painful as it is, can spark a desire to prevent others (or oneself) from going through the same loss [110]. The grief impacts patients but also underscores the necessity for a public health approach that includes screening, compassionate communication, and psychological support for healthy individuals, family members, and loved ones, as well as those with diminished health.

### 4.12. Limitations

Although this review employed structured methods, including the use of the PICO framework and predefined inclusion/exclusion criteria, it was not designed as a formal systematic review. As such, it was not registered in PROSPERO and does not include a PRISMA flowchart. The absence of Boolean operators and meta-analytic synthesis further distinguishes it from a systematic review. This approach was chosen to allow flexibility in exploring both qualitative and quantitative findings while maintaining methodological rigor.

## 5. Conclusions

In conclusion, this review emphasizes the importance of addressing both anticipatory and preparatory grief through targeted interventions, particularly psychotherapeutic support for cancer patients and their loved ones. The primary objective of understanding how both types of grief affect emotional and psychological well-being has been met, with findings showing that early psychological interventions focused on the patient, including therapy to process grief and develop coping mechanisms, are crucial for improving psychological resilience, reducing distress, and enhancing the overall quality of life. Regarding the secondary objective, the results underscore the need for interventions that empower coping strategies, encourage social interactions, and refer women to psychotherapy to help them process their feelings after a cancer diagnosis. Gender differences were also highlighted, revealing that women are more vulnerable to both anticipatory and preparatory grief. Future research should explore both clinical implications and protective factors to refine novel treatments and improve predictions of long-term outcomes. These feelings call for a public health approach supporting patients, the healthy, and their loved ones through screening, communication, and psychological care.

## Data Availability

No new data were created or analyzed in this study. Data sharing is not applicable to this article.

## Abbreviations

The following abbreviations are used in this manuscript:

PICO	Patient/population, Intervention, Comparison and Outcome
DALYs	Disability-adjusted life years
YLDs	years lived with a disability
PAP	Papanicolaou test
COVID-19	Coronavirus disease of 2019

## Appendix A

**Table A1:** The terminology within the literature on grief.

Term	Source	Definition
Bereavement	Shear, 2012 [81]	The psychological process and experience of letting go.
Grief	Shear, 2012 [81]	The reaction to bereavement, with an impact on feelings, thoughts, behaviors and physiological changes (varying in pattern and intensity over time). This is a highly unique and individual process.
Mourning	Shear, 2012 [81]	The process of integrating the consequences and finality of the loss into the memory system.
Complicated grief	Shear, 2012 [81]	Departure from the uncomplicated grief patterns with interruptions in the daily occupations due to the grief. Symptoms can include: - ruminations; - avoidance or compulsive proximity seeking; - inability to regulate emotions effectively. (For an extensive review and discussion on the ongoing debate about the syndrome of complicated grief, please see the Practice Pointer by Boelen and Smid) [82].
Anticipatory/preparatory grief	Fulton, 1980 [87]	The preparation for a prospective loss and a response to a loss of meaning and having to adjust the personal purpose and meaning in the own life. Characteristics: - mood fluctuations; - self-esteem is intact; - patient can enjoy seeing and interacting with friends and family; - able to experience pleasure; - able to look forward to special occasions.

**Table A2:** Guidelines for communication with cancer patients and their loved ones.

Questions Providing Guidance for Healthcare Professionals in Communicating with Terminally Ill Patients and Their Family Members/Caregivers	Multinational and Multi-Religious Perspectives on End-of-Life
Who should the doctor talk to first when discussing the test results or diagnosis?	In some cultures, the family members may have a greater say in decision making than the patient. It is important to respect and acknowledge family dynamics.
What are the cultural rituals for coping with dying person?	It is important for healthcare professionals to know how to address caregivers/family members and to what extent to share information regarding the status of the patient with each member of family/caregiver system.
What does the family consider to be the roles of each family member in handling the dying patient and death?	This is important in order to detect the best way of how to include the social support network.
What are the cultural rituals for handling the deceased person’s body?	In some religions, such as Buddhism, the body should not be touched for 3–8 h after breathing ceases as the spirit lingers on for some time. Furthermore, Hindus believe the body of the dead must be bathed, massaged in oils, dressed in new clothes, and then cremated before the next sunrise.
What are the final arrangements for the body and honouring the death?	It is important for any medical institution to facilitate the funeral procedures and support the family/caregivers by decreasing the burden of the painful organizational process.
What are the family’s beliefs about what happens after death?	Some religions cope with death and dying more easily, e.g., both Muslims and Christians believe in an afterlife and view the earthly life more in terms of preparing for an eternal life. In the Jewish tradition, the focus is on the purpose of an earthly life, which is to fulfil one’s duties to god and one’s fellow men. This may introduce difference in grieving intensity.
Are certain types of dying/death less acceptable for certain religions?	Consider for example suicide, euthanasia or palliative sedation.
Is organ donation allowed within the patient’s religion?	This is important, because some religion do not agree with organ donation.
What is the preferred manner of the funeral within the patients religion?	In case the patient does not have anyone and needs to be taken care of by a medical institution after the death, it is important to be aware of the fact that some religions favour cremation over burial.

**Table A3:** Applying psychotherapeutic interventions against preparatory and anticipatory grieving: how it helps the individual with cancer, the patient’s loved ones, clinical healing, and clinicians.

Preparatory and Anticipatory Grief Psychotherapy	How It Helps the Individual with Cancer	How It Helps the Patient’s Loved Ones	How It Helps Clinical Healing and/or Clinician
**Emotional** **Processing**	Helps patients confront and articulate their fears, reducing emotional distress.	Assists family members in understanding the patient’s emotions and responding empathetically.	Enhances the therapeutic alliance and improves communication between clinicians and patients.
**Coping Strategies**	Provides patients with effective coping mechanisms to manage grief and stress.	Empowers loved ones with strategies to support the patient and manage their own emotions.	Reduces the likelihood of burnout by equipping patients and families with practical coping tools.
**Family** **Involvement**	Engages family members in the care process, improving patient support.	Facilitates family cohesion and understanding, which can alleviate relational stress.	Strengthens the support network around the patient, contributing to a more comprehensive care approach.
**Preparation for Future Loss**	Allows patients to make practical and emotional preparations, providing a sense of control.	Helps loved ones anticipate and prepare for future grief, potentially reducing their emotional burden.	Aids clinicians in managing end-of-life care more effectively by aligning patient and family expectations.
**Reduced** **Emotional** **Distress**	Leads to decreased levels of anxiety and depression related to the diagnosis.	Mitigates secondary stress and anticipatory grief experienced by family members.	Supports clinicians in providing effective care without being overwhelmed by the emotional complexity of the case.

**Table A4:** Comparison between anticipatory grief and preparatory grief, along with a brief section on how illness representations influence coping strategies.

Aspect	Anticipatory Grief	Preparatory Grief
**Definition**	Grief experienced by individuals expecting to lose a loved one (pre-loss)	Grief experienced by individuals nearing the end of their own life (post-loss preparations)
**Who Experiences It**	Family members, caregivers, and loved ones of someone with a terminal illness	The person who is terminally ill, as they prepare for their own death
**Emotional Response**	Feelings of sadness, fear, anxiety, and uncertainty about the impending loss	Feelings of sadness, existential anxiety, mortality awareness, and emotional distress related to one’s own death
**Focus**	Focus on the upcoming loss of a loved one and emotional preparedness	Focus on self-reflection, the impact on loved ones, and managing end-of-life issues
**Common Symptoms**	Anxiety, depression, anticipatory grief symptoms, heightened emotional distress	Sadness, fear, anger, acceptance, spiritual concerns, and emotional withdrawal
**Coping Strategies**	Emotional regulation, preparing for life without the loved one, practical planning	Reflection on life, closure, legacy-building, preparing loved ones emotionally and practically
**Impact on Quality of Life**	Can negatively affect well-being due to ongoing emotional strain and uncertainty	Can reduce quality of life by focusing on mortality and what’s to come
**Psychotherapeutic Interventions**	Counseling, therapy focusing on emotional expression and practical preparation for the loss	Supportive therapy focusing on life review, existential concerns, emotional support for family, and closure
**Illness Representations**	Patients and families may struggle with understanding the illness trajectory, leading to greater uncertainty and distress	Patients who have a clear understanding of their illness may engage in more active preparations, while those with misconceptions or denial may experience more prolonged emotional distress
**Coping Influence by** **Illness Representation**	Illness representations, such as viewing the illness as uncontrollable or as a threat, often lead to heightened emotional distress and avoidance behaviors in coping	A more accurate or accepted illness representation may help in managing anticipatory grief by fostering acceptance and active preparation for death, but denial or unrealistic expectations can increase distress
**Gender Differences**	Women may experience greater emotional intensity, leading to increased vulnerability to anticipatory grief	Women may be more likely to focus on relationships and caregiving aspects, leading to more emotional and social preparation for their death

## References

[1] Bray F., Laversanne M., Sung H., Ferlay J., Siegel R.L., Soerjomataram I., Jemal A. (2024). Global cancer statistics 2022: GLOBOCAN estimates of incidence and mortality worldwide for 36 cancers in 185 countries. CA Cancer J. Clin..

[2] GBD 2021 Diseases and Injuries Collaborators (2024). Global incidence, prevalence, years lived with disability (YLDs), disability-adjusted life-years (DALYs), and healthy life expectancy (HALE) for 371 diseases and injuries in 204 countries and territories and 811 subnational locations, 1990–2021: A systematic analysis for the Global Burden of Disease Study 2021. Lancet.

[3] Australian Institute of Health and Welfare, Australian Government Department of Health (2016). Analysis of colorectal cancer outcomes for the Australian National Bowel Cancer Screening Program. Asia-Pac. J. Clin. Oncol..

[4] Zorzi M., Urso E.D.L. (2023). Impact of colorectal cancer screening on incidence, mortality and surgery rates: Evidences from programs based on the fecal immunochemical test in Italy. Dig. Liver Dis..

[5] Massat N.J., Dibden A., Parmar D., Cuzick J., Sasieni P.D., Duffy S.W. (2016). Impact of Screening on Breast Cancer Mortality: The UK Program 20 Years On. Cancer Epidemiol. Biomark. Prev..

[6] Guthmuller S., Carrieri V., Wübker A. (2023). Effects of organized screening programs on breast cancer screening, incidence, and mortality in Europe. J. Health Econ..

[7] Živković Perišić S., Miljuš D., Jovanović V. (2021). Registracija malignih bolesti u proceni opterećenosti društva rakom. Glas. Javnog Zdr..

[8] Sekulić M., Milosavljević M., Jovanović V. (2025). Značaj sprovođenja promotivnih aktivnosti putem sredstava javnog informisanja u prevenciji raka grlića materice. Glas. Javnog Zdr..

[9] Sarma E.A., Silver M.I., Kobrin S.C., Marcus P.M., Ferrer R.A. (2019). Cancer screening: Health impact, prevalence, correlates, and interventions. Psychol. Health.

[10] Fujimori M., Uchitomi Y. (2009). Preferences of cancer patients regarding communication of bad news: A systematic literature review. Jpn. J. Clin. Oncol..

[11] Coelho A., de Brito M., Teixeira P., Frade P., Barros L., Barbosa A. (2020). Family Caregivers’ Anticipatory Grief: A Conceptual Framework for Understanding Its Multiple Challenges. Qual. Health Res..

[12] Sikstrom L., Saikaly R., Ferguson G., Mosher P.J., Bonato S., Soklaridis S. (2019). Being there: A scoping review of grief support training in medical education. PLoS ONE.

[13] Esplen M.J., Wong J., Vachon M.L.S., Leung Y. (2022). A Continuing Educational Program Supporting Health Professionals to Manage Grief and Loss. Curr. Oncol..

[14] National Cancer Institute of U.S. Department of Health and Human Services http://www.cancer.gov/publications/dictionaries/cancer-terms?CdrID=46053.

[15] National Cancer Institute of U.S. Department of Health and Human Services https://www.cancer.gov/about-cancer/coping.

[16] Al-Gamal E. (2013). Quality of life and anticipatory grieving among parents living with a child with cerebral palsy. Int. J. Nurs. Pract..

[17] Shear M.K., Ghesquiere A., Glickman K. (2013). Bereavement and complicated grief. Curr. Psychiatry Rep..

[18] International Agency for Research on Cancer GLOBOCAN 2012 v1.0, Cancer Incidence and Mortality Worldwide: IARC CancerBase No. 11. https://publications.iarc.fr/Databases/Iarc-Cancerbases/GLOBOCAN-2012-Estimated-Cancer-Incidence-Mortality-And-Prevalence-Worldwide-In-2012-V1.0-2012.

[19] Cancer Research UK (UK CR) Worldwide Cancer Incidence 2012. https://www.cancerresearchuk.org/health-professional/cancer-statistics/worldwide-cancer/incidence#ref-1.

[20] Dorak M.T., Karpuzoglu E. (2012). Gender differences in cancer susceptibility: An inadequately addressed issue. Front. Genet..

[21] Global Burden of Disease Cancer Collaboration (2017). Global, regional, and national cancer incidence, mortality, years of life lost, years lived with disability, and disability-adjusted life-years for 32 cancer groups, 1990 to 2015: A systematic analysis for the Global Burden of Disease Study. JAMA Oncol..

[22] Woo Y.L., Kyrgiou M., Bryant A., Everett T., Dickinson H.O. (2012). Centralisation of services for gynaecological cancer. Cochrane Database Syst. Rev..

[23] Wu M.-M., Chen H.-C., Chen C.-L., You S.-L., Cheng W.-F., Chen C.-A., Lee T.-C., Chen C.-J. (2014). A prospective study of gynecological cancer risk in relation to adiposity factors: Cumulative incidence and association with plasma adipokine levels. PLoS ONE.

[24] He Y., Su Y., Zeng J., Chong W., Hu X., Zhang Y., Peng X. (2022). Cancer-specific survival after diagnosis in men versus women: A pan-cancer analysis. MedComm.

[25] Brown L.F., Kroenke K., Theobald D.E., Wu J., Tu W. (2010). The association of depression and anxiety with health-related quality of life in cancer patients with depression and/or pain. Psycho-Oncology.

[26] Stark D., Kiely M., Smith A., Velikova G., House A., Selby P. (2002). Anxiety disorders in cancer patients: Their nature, associations, and relation to quality of life. J. Clin. Oncol..

[27] Saavedra Pérez H.C., Direk N., Milic J., Ikram M.A., Hofman A., Tiemeier H. (2017). The Impact of Complicated Grief on Diurnal Cortisol Levels Two Years After Loss: A Population-Based Study. Psychosom. Med..

[28] Vargas K.G., Milic J., Zaciragic A., Wen K., Jaspers L., Nano J., Dhana K., Bramer W.M., Kraja B., Van Beeck E. (2016). The functions of estrogen receptor beta in the female brain: A systematic review. Maturitas.

[29] Zaider T., Kissane D. (2009). The assessment and management of family distress during palliative care. Curr. Opin. Support. Palliat. Care.

[30] Arantzamendi M., Sapeta P., Belar A., Centeno C. (2024). How palliative care professionals develop coping competence through their career: A grounded theory. Palliat. Med..

[31] Singer J., Roberts K.E., McLean E., Fadalla C., Coats T., Rogers M., Wilson M.K., Godwin K., Lichtenthal W.G. (2022). An examination and proposed definitions of family members’ grief prior to the death of individuals with a life-limiting illness: A systematic review. Palliat. Med..

[32] Masterson M.P., Schuler T.A., Kissane D.W. (2013). Family focused grief therapy: A versatile intervention in palliative care and bereavement. Bereave Care.

[33] Szuhany K.L., Malgaroli M., Miron C.D., Simon N.M. (2021). Prolonged Grief Disorder: Course, Diagnosis, Assessment, and Treatment. Focus.

[34] Cheung J.T.K., Au D., Ip A.H.F., Chan J., Ng K., Cheung L., Yuen J., Hui E., Lee J., Lo R. (2020). Barriers to advance care planning: A qualitative study of seriously ill Chinese patients and their families. BMC Palliat. Care.

[35] Vergo M.T., Whyman J., Li Z., Kestel J., James S.L., Rector C., Salsman J.M. (2017). Assessing Preparatory Grief in Advanced Cancer Patients as an Independent Predictor of Distress in an American Population. J. Palliat. Med..

[36] Richardson P. (2014). Spirituality, religion and palliative care. Ann. Palliat. Med..

[37] Tavares A.P., Martins H., Pinto S., Caldeira S., Pontífice Sousa P., Rodgers B. (2022). Spiritual comfort, spiritual support, and spiritual care: A simultaneous concept analysis. Nurs. Forum.

[38] Zheng R., Guo Q., Chen Z., Zeng Y. (2023). Dignity therapy, psycho-spiritual well-being and quality of life in the terminally ill: Systematic review and meta-analysis. BMJ Support. Palliat. Care.

[39] Hopf D., Eckstein M., Aguilar-Raab C., Warth M., Ditzen B. (2020). Neuroendocrine mechanisms of grief and bereavement: A systematic review and implications for future interventions. J. Neuroendocrinol..

[40] Kaltman S., Bonanno G.A. (2003). Trauma and bereavement: Examining the impact of sudden and violent deaths. J. Anxiety Disord..

[41] Schneiderman N., Ironson G., Siegel S.D. (2005). Stress and health: Psychological, behavioral, and biological determinants. Annu. Rev. Clin. Psychol..

[42] Davis M.T., Holmes S.E., Pietrzak R.H., Esterlis I. (2017). Neurobiology of Chronic Stress-Related Psychiatric Disorders: Evidence from Molecular Imaging Studies. Chronic Stress.

[43] Kuhney F.S., Miklowitz D.J., Schiffman J., Mittal V.A. (2023). Family-Based Psychosocial Interventions for Severe Mental Illness: Social Barriers and Policy Implications. Policy Insights Behav. Brain Sci..

[44] Zhang D., Lee E.K.P., Mak E.C.W., Ho C.Y., Wong S.Y.S. (2021). Mindfulness-based interventions: An overall review. Br. Med. Bull..

[45] Varghese M., Kirpekar V., Loganathan S. (2020). Family Interventions: Basic Principles and Techniques. Indian J. Psychiatry.

[46] Pun J., Chow J.C.H., Fok L., Cheung K.M. (2023). Role of patients’ family members in end-of-life communication: An integrative review. BMJ Open.

[47] Di Nota P.M., Bahji A., Groll D., Carleton R.N., Anderson G.S. (2021). Proactive psychological programs designed to mitigate posttraumatic stress injuries among at-risk workers: A systematic review and meta-analysis. Syst. Rev..

[48] Biswas J., Drogin E.Y., Gutheil T.G. (2018). Treatment delayed is treatment denied. J. Am. Acad. Psychiatry Law Online.

[49] Crowley A., Kirst M. (2025). The Experiences of Virtual Support for Grief and Bereavement. Illn. Crisis Loss.

[50] Lundorff M., Bonanno G.A., Johannsen M., O’Connor M. (2020). Are there gender differences in prolonged grief trajectories? A registry-sampled cohort study. J. Psychiatr. Res..

[51] Kelly M.M., Tyrka A.R., Price L.H., Carpenter L.L. (2008). Sex differences in the use of coping strategies: Predictors of anxiety and depressive symptoms. Depress. Anxiety.

[52] Sharma N., Chakrabarti S., Grover S. (2016). Gender differences in caregiving among family—Caregivers of people with mental illnesses. World J. Psychiatry.

[53] Stroebe M., Stroebe W., Schut H. (2001). Gender Differences in Adjustment to Bereavement: An Empirical and Theoretical Review. Rev. Gen. Psychol..

[54] Givon E., Berkovich R., Oz-Cohen E., Rubinstein K., Singer-Landau E., Udelsman-Danieli G., Meiran N. (2023). Are women truly “more emotional” than men? Sex differences in an indirect model-based measure of emotional feelings. Curr. Psychol..

[55] Pătrașcu C., Oroian B.A., Soveja A., Marusic R.I., Cozmin M., Crețu O., Nechita P. (2024). Gender differences in depression: Symptoms, causes and treatments. Bull. Integr. Psychiatry.

[56] Ellemers N. (2018). Gender stereotypes. Annu. Rev. Psychol..

[57] Chad-Friedman E., Coleman S., Traeger L.N., Pirl W.F., Goldman R., Atlas S.J., Park E.R. (2017). Psychological distress associated with cancer screening: A systematic review. Cancer.

[58] Hasdenteufel M., Quintard B. (2022). Psychosocial factors affecting the bereavement experience of relatives of palliative-stage cancer patients: A systematic review. BMC Palliat. Care.

[59] El-Jawahri A., Greer J.A., Park E.R., Jackson V.A., Kamdar M., Rinaldi S.P., Gallagher E.R., Jagielo A.D., Topping C.E.W., Elyze M. (2021). Psychological Distress in Bereaved Caregivers of Patients with Advanced Cancer. J. Pain Symptom Manag..

[60] Djordjevic S., Boricic K., Radovanovic S., Simic Vukomanovic I., Mihaljevic O., Jovanovic V. (2024). Demographic and socioeconomic factors associated with cervical cancer screening among women in Serbia. Front. Public Health.

[61] Djordjević S., Dimitrijev I., Boričić K., Radovanović S., Vukomanović I.S., Mihaljević O., Jovanović S., Randjelović N., Lacković A., Knezević S. (2024). Sociodemographic Factors Associated with Breast Cancer Screening among Women in Serbia, National Health Survey. Iran. J. Public Health.

[62] Collins R.E., Lopez L.M., Marteau T.M. (2011). Emotional impact of screening: A systematic review and meta-analysis. BMC Public Health.

[63] Doherty J., Cullen S., Casey B., Lloyd B., Sheehy L., Brosnan M., Barry T., McMahon A., Coughlan B. (2018). Bereavement care education and training in clinical practice: Supporting the development of confidence in student midwives. Midwifery.

[64] Shear M.K., Muldberg S., Periyakoil V. (2017). Supporting patients who are bereaved. BMJ.

[65] Lazarus R.S. (1993). Coping theory and research: Past, present, and future. Psychosom. Med..

[66] O’Brien C.W., Moorey S. (2010). Outlook and adaptation in advanced cancer: A systematic review. Psycho-Oncology.

[67] Deimling G.T., Wagner L.J., Bowman K.F., Sterns S., Kercher K., Kahana B. (2006). Coping among older-adult, long-term cancer survivors. Psycho-Oncology.

[68] Edwards B., Clarke V. (2004). The psychological impact of a cancer diagnosis on families: The influence of family functioning and patients’ illness characteristics on depression and anxiety. Psycho-Oncology.

[69] Singer S., Das-Munshi J., Brahler E. (2010). Prevalence of mental health conditions in cancer patients in acute care: A meta-analysis. Ann. Oncol..

[70] Stanton A.L., Danoff-Burg S., Huggins M.E. (2002). The first year after breast cancer diagnosis: Hope and coping strategies as predictors of adjustment. Psycho-Oncology.

[71] Kvillemo P., Branstrom R. (2014). Coping with breast cancer: A meta-analysis. PLoS ONE.

[72] Mystakidou K., Tsilika E., Parpa E., Katsouda E., Sakkas P., Galanos A., Vlahos L. (2006). Demographic and clinical predictors of preparatory grief in a sample of advanced cancer patients. Psycho-Oncology.

[73] Sanders S., Adams K.B. (2005). Grief reactions and depression in caregivers of individuals with Alzheimer’s disease: Results from a pilot study in an urban setting. Health Soc. Work.

[74] Stanton A.L., Snider P.R. (1993). Coping with a breast cancer diagnosis: A prospective study. Health Psychol..

[75] Hack T.F., Degner L.F. (2004). Coping responses following breast cancer diagnosis predict psychological adjustment three years later. Psycho-Oncology.

[76] Wolberg W.H., Romsaas E.P., Tanner M.A., Malec J.F. (1989). Psychosexual adaptation to breast cancer surgery. Cancer.

[77] Burwell S.R., Case L.D., Kaelin C., Avis N.E. (2006). Sexual problems in younger women after breast cancer surgery. J. Clin. Oncol..

[78] Arroyo J.M., Lopez M.L. (2011). Psychological problems derived from mastectomy: A qualitative study. Int. J. Surg. Oncol..

[79] Wilkes S., Coulson S., Crosland A., Rubin G., Stewart J. (2010). Experience of fertility preservation among younger people diagnosed with cancer. Hum. Fertil..

[80] O’Connor M., O’Leary E., Waller J., Gallagher P., D’Arcy T., Flannelly G., Martin C.M., McRae J., Prendiville W., Ruttle C. (2016). Trends in, and predictors of, anxiety and specific worries following colposcopy: A 12-month longitudinal study. Psycho-Oncology.

[81] Shear M.K. (2012). Getting straight about grief. Depress. Anxiety.

[82] Boelen P.A., Smid G.E. (2017). Disturbed grief: Prolonged grief disorder and persistent complex bereavement disorder. BMJ.

[83] Milic J., Saavedra Perez H., Zuurbier L.A., Boelen P.A., Rietjens J.A., Hofman A., Tiemeier H. (2017). The Longitudinal and Cross-Sectional Associations of Grief and Complicated Grief With Sleep Quality in Older Adults. Behav. Sleep Med..

[84] Maercker A., Brewin C.R., Bryant R.A., Cloitre M., van Ommeren M., Jones L.M., Humayan A., Kagee A., Llosa A.E., Rousseau C. (2013). Diagnosis and classification of disorders specifically associated with stress: Proposals for ICD-11. World Psychiatry.

[85] Prigerson H.G., Horowitz M.J., Jacobs S.C., Parkes C.M., Aslan M., Goodkin K., Raphael B., Marwit S.J., Wortman C., Neimeyer R.A. (2009). Prolonged grief disorder: Psychometric validation of criteria proposed for DSM-V and ICD-11. PLoS Med..

[86] Milic J., Muka T., Ikram M.A., Franco O.H., Tiemeier H. (2017). Determinants and Predictors of Grief Severity and Persistence: The Rotterdam Study. J. Aging Health.

[87] Fulton R., Gottesman D.J. (1980). Anticipatory grief: A psychosocial concept reconsidered. Br. J. Psychiatry.

[88] Kübler-Ross E. (1969). On Death and Dying.

[89] Fulton G., Madden C., Minichiello V. (1996). The social construction of anticipatory grief. Soc. Sci. Med..

[90] Cheng J.O., Lo R.S., Chan F.M., Kwan B.H., Woo J. (2010). An exploration of anticipatory grief in advanced cancer patients. Psycho-Oncology.

[91] Leventhal H., Diefenbach M., Leventhal E.A. (1992). Illness cognition: Using common sense to understand treatment adherence and affect cognition interactions. Cogn. Ther. Res..

[92] Heijmans M. (1999). The role of patients’ illness representations in coping and functioning with Addison’s disease. Br. J. Health Psychol..

[93] Gökler-Danışman I., Yalçınay-İnan M., Yiğit İ. (2017). Experience of grief by patients with cancer in relation to perceptions of illness: The mediating roles of identity centrality, stigma-induced discrimination, and hopefulness. J. Psychosoc. Oncol..

[94] Husson O., Thong M.S., Mols F., Oerlemans S., Kaptein A.A., van de Poll-Franse L.V. (2013). Illness perceptions in cancer survivors: What is the role of information provision?. Psycho-Oncology.

[95] Krishnasamy M. (1996). Social support and the patient with cancer: A consideration of the literature. J. Adv. Nurs..

[96] Toyama H., Honda A. (2016). Using Narrative Approach for Anticipatory Grief Among Family Caregivers at Home. Glob. Qual. Nurs. Res..

[97] Overton B.L., Cottone R.R. (2016). Anticipatory Grief: A Family Systems Approach. Fam. J..

[98] Lang-Rollin I., Berberich G. (2018). Psycho-oncology. Dialog. Clin. Neurosci..

[99] Bultz B.D., Travado L., Jacobsen P.B., Turner J., Borras J.M., Ullrich A.W. (2015). 2014 President’s plenary international psycho-oncology society: Moving toward cancer care for the whole patient. Psycho-Oncology.

[100] Mao J.J., Pillai G.G., Andrade C.J., Ligibel J.A., Basu P., Cohen L., Khan I.A., Mustian K.M., Puthiyedath R., Dhiman K.S. (2022). Integrative oncology: Addressing the global challenges of cancer prevention and treatment. CA Cancer J. Clin..

[101] Carlson L.E. (2023). Psychosocial and integrative oncology: Interventions across the disease trajectory. Annu. Rev. Psychol..

[102] Thakur M., Mishra A.K., Gajera G. (2025). Anxiety and Depression Among Breast Cancer Survivors: A Trauma Beyond Cancer. Handbook of the Behavior and Psychology of Disease.

[103] Ajluni V. (2025). Integrating psychiatry and family medicine in the management of somatic symptom disorders: Diagnosis, collaboration, and communication strategies. J. Gen. Fam. Med..

[104] Ibrahim A.M., Wahba N.M.I., Zaghamir D.E.F., Mersal N.A., Mersal F.A., Ali R.A.E.S., Eltaib F.A., Mohamed H.A.H. (2024). Impact of a comprehensive rehabilitation palliative care program on the quality of life of patients with terminal cancer and their informal caregivers: A quasi-experimental study. BMC Nurs..

[105] Sebri V., Pravettoni G. (2023). Tailored psychological interventions to manage body image: An opinion study on breast cancer survivors. Int. J. Environ. Res. Public Health.

[106] Lyu X.C., Jiang H.J., Lee L.H., Yang C.I., Sun X.Y. (2024). Oncology nurses’ experiences of providing emotional support for cancer patients: A qualitative study. BMC Nurs..

[107] Becqué Y.N., van der Wel M., Aktan-Arslan M., Driel A.G.V., Rietjens J.A., van der Heide A., Witkamp E. (2023). Supportive interventions for family caregivers of patients with advanced cancer: A systematic review. Psycho-Oncology.

[108] Chia K.H. (2024). The Psychosocial Developmental Phases of Death (Mortality) Awareness: What Educational Therapists should know when working with Terminally Ill Clients. Asian J. Interdiscip. Res..

[109] Hamdoun S., Monteleone R., Bookman T., Michael K. (2023). AI-based and digital mental health apps: Balancing need and risk. IEEE Technol. Soc. Mag..

[110] Lichtenthal W.G., Roberts K.E., Donovan L.A., Breen L.J., Aoun S.M., Connor S.R., Rosa W.E. (2024). Investing in bereavement care as a public health priority. Lancet Public Health.

